# Flow cytometric micronucleus assay and TGx-DDI transcriptomic biomarker analysis of ten genotoxic and non-genotoxic chemicals in human HepaRG™ cells

**DOI:** 10.1186/s41021-019-0139-2

**Published:** 2020-02-04

**Authors:** Julie K. Buick, Andrew Williams, Rémi Gagné, Carol D. Swartz, Leslie Recio, Stephen S. Ferguson, Carole L. Yauk

**Affiliations:** 1 Environmental Health Science and Research Bureau, Health Canada, Ottawa, Ontario K1A 0K9 Canada; 2 Integrated Laboratory Systems Inc. (ILS), Research Triangle Park, Durham, North Carolina 27709 USA; 3 National Toxicology Program, National Institute of Environmental Health Sciences, Research Triangle Park, Durham, North Carolina 27709 USA; 4 Health Canada, Environmental Health Centre, 50 Colombine Driveway, PL 0803A, Ottawa, Ontario K1A 0K9 Canada

**Keywords:** Genetic toxicology, TGx-28.65 genomic biomarker, RNA-Seq, Micronucleus, Toxicogenomics

## Abstract

**Background:**

Modern testing paradigms seek to apply human-relevant cell culture models and integrate data from multiple test systems to accurately inform potential hazards and modes of action for chemical toxicology. In genetic toxicology, the use of metabolically competent human hepatocyte cell culture models provides clear advantages over other more commonly used cell lines that require the use of external metabolic activation systems, such as rat liver S9. HepaRG™ cells are metabolically competent cells that express Phase I and II metabolic enzymes and differentiate into mature hepatocyte-like cells, making them ideal for toxicity testing. We assessed the performance of the flow cytometry in vitro micronucleus (MN) test and the TGx-DDI transcriptomic biomarker to detect DNA damage-inducing (DDI) chemicals in human HepaRG™ cells after a 3-day repeat exposure. The biomarker, developed for use in human TK6 cells, is a panel of 64 genes that accurately classifies chemicals as DDI or non-DDI. Herein, the TGx-DDI biomarker was analyzed by Ion AmpliSeq whole transcriptome sequencing to assess its classification accuracy using this more modern gene expression technology as a secondary objective.

**Methods:**

HepaRG™ cells were exposed to increasing concentrations of 10 test chemicals (six genotoxic chemicals, including one aneugen, and four non-genotoxic chemicals). Cytotoxicity and genotoxicity were measured using the In Vitro MicroFlow® kit, which was run in parallel with the TGx-DDI biomarker.

**Results:**

A concentration-related decrease in relative survival and a concomitant increase in MN frequency were observed for genotoxic chemicals in HepaRG™ cells. All five DDI and five non-DDI agents were correctly classified (as genotoxic/non-genotoxic and DDI/non-DDI) by pairing the test methods. The aneugenic agent (colchicine) yielded the expected positive result in the MN test and negative (non-DDI) result by TGx-DDI.

**Conclusions:**

This next generation genotoxicity testing strategy is aligned with the paradigm shift occurring in the field of genetic toxicology. It provides mechanistic insight in a human-relevant cell-model, paired with measurement of a conventional endpoint, to inform the potential for adverse health effects. This work provides support for combining these assays in an integrated test strategy for accurate, higher throughput genetic toxicology testing in this metabolically competent human progenitor cell line.

## Introduction

Twenty-first century toxicology necessitates alternative test methods that are more efficient and effective to evaluate the backlog of chemicals requiring assessment [1–6]. Thus, higher throughput, higher content tests in human and animal cell cultures are being investigated for this purpose [7–11]. One important key to the improvement of in vitro testing strategies is the use of relevant human cell culture systems that exhibit intact intracellular compartmentalization and model tissue-like functionality dynamics (e.g., in vivo metabolic processes and toxicological effects). In parallel, modern testing strategies rely more heavily on the measurement of mechanistic changes that inform the potential for adverse outcomes in humans [12–16]. An ideal twenty-first century strategy should apply high-content and higher-throughput approaches to efficiently leverage mechanistic information to predict apical effects and inform mode of action (MOA).

Genotoxicity testing is an imperative component of chemical risk assessment, as damage to genetic material leading to mutations, chromosome damage, or genetic instability can result in hereditary diseases and cancer [17, 18]. Historically, in vitro genotoxicity tests have been performed in rodent cell lines such as CHO, V79, CHL, and L5178Y, in addition to human TK6 lymphoblastoid cells and peripheral blood lymphocytes [19, 20]. These cell types have various limitations, a significant one being their lack of xenobiotic metabolism required for both activation and detoxification [21]. Thus, these cell culture models require the use of exogenous metabolic activating systems (i.e., typically induced rat liver S9 subcellular fractions supplemented with NADPH or a NADPH generating system to support cytochrome P450 (CYP450) activity for pro-mutagen activation). The addition of S9 can be problematic in that it can cause cytotoxicity, models highly-induced CYP450-mediated rat liver metabolism, generally lacks support of Phase II metabolism pathways, may require optimization of the amount and type of induction for bioactivation of certain chemicals, and the efficiency can vary between lots [22–29]. Furthermore, it is important to ensure the cell model is relevant to humans. While the “gold standard” for in vitro modeling of human liver functionality (e.g., liver enzyme induction, biliary efflux transport) has been the culture of primary human hepatocytes (PHHs), given their retention of metabolic enzyme expression and proficiency for hepatic receptor signalling pathways, PHHs can be phenotypically unstable over time with differentiation that rapidly diminishes ex vivo [30–32]. Moreover, profound donor-to-donor variability paired with the finite number of cells available from an individual liver limits their broader use in year-over-year screening platforms. Thus, a next generation in vitro testing strategy would benefit from the use of human-relevant cell models with metabolic capabilities that more effectively mimic in vivo metabolism without the potential complications and limitations of exogenous S9 addition or the use of PHHs [30, 33–35].

Human HepaRG™ cells are gaining more traction as a cell line of choice for in vitro testing [33, 35–37]. These cells, derived from a hepatocellular carcinoma in a Caucasian female, differentiate into mature co-cultures of hepatocyte- and cholangiocyte-like cells and express relevant levels of Phase I and Phase II metabolic enzymes, transporters and nuclear receptors, making them ideal for year-over-year drug metabolism and toxicity screening while overcoming the limitations of PHHs in culture [30, 32–35]. Cryopreserved HepaRG™ cells have also been extensively validated for in vitro cytochrome P450 induction and have been determined to be a reliable metabolically competent human cell line that can be used as a replacement for PHHs [38]. There are now hundreds of research publications using fresh or cryopreserved HepaRG™ cells that have studied chemically-induced responses to hepatic pathways at the molecular level [39, 40]. There is also great interest in the use of HepaRG™ cells in genetic toxicology testing. Indeed, the flow cytometry-based micronucleus (MN) assay has been adapted for use with HepaRG™ cells [41, 42], and a variety of investigators have used this progenitor cell line for the assessment of chemically-induced genetic effects [32, 36, 43–48].

Various studies have shown that HepaRG™ transcriptional profiles are more similar to PHHs than other commonly used liver cell lines (e.g., HepG2), supporting their use as a human liver model for chemically-induced gene expression responses for hazard identification and the evaluation of genotoxic potential [32, 39, 44]. Moreover, several studies have demonstrated the ability to use transcriptional profiling in HepaRG™ cells to differentiate genotoxic from non-genotoxic carcinogens and non-carcinogens, and that the classification accuracy is higher in HepaRG™ cells than in other in vitro liver models [46, 47, 49]. Furthermore, recent work has shown that chemically-induced transcriptional responses in HepaRG™ cells can be measured in a high-throughput manner using TempO-Seq® as an effective in vitro tool to study toxicological responses [40, 50]. Overall, these studies provide a strong rationale for the use of the liver-based HepaRG™ model paired with transcriptomic analysis using various gene expression technologies as a strategy to identify genotoxic chemicals and their mechanism of action for chemical evaluation.

The overarching objective of the present study was to explore the use of HepaRG™ cells in genetic toxicology testing using the flow cytometry MN assay and the TGx-DDI transcriptomic biomarker assay. To do this, HepaRG™ cell cultures were exposed to ten test chemicals (six genotoxic chemicals, including one aneugen, and four non-genotoxic chemicals) to evaluate the performance of these assays together in these cells. A secondary objective was to explore the performance of the TGx-DDI transcriptomic biomarker analyzed using AmpliSeq, an RNA-sequencing based technology. Furthermore, by combining a validated genotoxicity test (e.g., the MN test) with a novel genotoxicity test that provides mechanistic data (e.g., TGx-DDI), there is the added benefit of utilizing information that is currently lacking from the standard genotoxicity test paradigm for predictive purposes and to gain insight into genotoxic MOAs.

The TGx-DDI biomarker was developed and is undergoing validation through the Health and Environmental Sciences Institute’s (HESI) Technical Committee for Emerging Systems Toxicology for the Assessment of Risk (eSTAR), as a transcriptomic approach to predict the DNA damaging potential of chemicals [51, 52]. Transcriptional changes in the 64 genes that comprise the TGx-DDI are used to classify compounds as DNA damage-inducing (DDI) and non-DDI in human lymphoblastoid TK6 cells in the presence/absence of S9 metabolic activation using Agilent gene expression DNA microarrays [53, 54]. The biomarker has been demonstrated to improve upon the problems associated with the low specificity of existing in vitro chromosome damage assays [52]. Moreover, the biomarker informs that the observation of chromosomal changes (e.g., in the MN assay) are the result of DNA damage (to differentiate from aneugenic mechanisms) and that the damage was sufficient to induce a robust change in the transcription of p53-regulated genes. The present study investigates the performance of the TGx-DDI biomarker in HepaRG™ cells using RNA-Seq (a more precise, modern transcriptional profiling approach). The work explores the accuracy of TGx-DDI predictions in HepaRG™ cells relative to published information on the test chemicals used in the experiment, and through comparison with concurrent results with the MN assay, a validated regulatory assay to assess chromosomal aberrations and aneugenicity.

We exposed HepaRG™ cells to the ten test chemicals (Table 1) at six different concentrations in a repeated exposure study design. The DDI chemicals in this study are: aflatoxin B1 (AFB1), cisplatin (CISP), etoposide (ETP), methyl methanesulfonate (MMS), and 2-nitrofluorene (2-NF). These DDI chemicals exert their genotoxic effects through various mechanisms, including the formation of bulky adducts (AFB1, 2-NF), alkylation of DNA (MMS), the creation of DNA cross-links (CISP), and topoisomerase II inhibition (ETP). The non-DDI chemicals are: ampicillin trihydrate (AMP), colchicine (COL), 2-deoxy-D-glucose (2DG), sodium ascorbate (ASC), and sodium chloride (NaCl). The non-DDI test chemicals encompass an antibiotic (AMP), an antimitotic agent that is well-known to cause aneuploidy (COL), a glycolysis inhibitor (2DG), a mineral salt of ascorbic acid (ASC), and salt (NaCl). The in vitro MicroFlow® kit was applied to measure cytotoxicity and MN frequency. In parallel, high-throughput Ion AmpliSeq Human Transcriptome sequencing technology with an Ion Proton sequencer was used to measure gene expression. AmpliSeq is a targeted, whole transcriptome profiling approach that enables the concurrent measurement of more than 20,000 human genes [55]. By pairing a sensitive modern chromosome damage test (i.e., the MN test – considered a gold standard within this study) with mechanistic data (i.e., TGx-DDI) in this human-relevant, metabolically competent cell line, this novel test strategy is expected to yield results that are likely to be relevant to humans and positive findings should be high priority for in vivo testing.

**Table 1:** Test Chemical Information

Test Chemical	Chemical Abbreviation	CAS No.	Vehicle Control	Concentrations Tested
DNA Damage-Inducing (DDI) Chemicals				
Aflatoxin B1	AFB1	1162-65-8	DMSO	0.1, 0.25, 0.5, 1, 2.5, 5* μM
Cisplatin	CISP	15,663–27-1	DMSO	1, 2, 3, 5, 10, 20* μM
Etoposide	ETP	33,419–42-0	DMSO	0.25, 0.5, 1, 2.5, 5, 10 μM
Methyl methanesulfonate	MMS	66–27-3	Water	5, 10, 20, 50, 100, 200 μM
2-Nitrofluorene	2NF	607–57-8	DMSO	2, 10, 50, 100, 250, 500* μM
Non-DNA Damage-Inducing (Non-DDI) Chemicals				
Ampicillin Trihydrate	AMP	7177-48-2	Media	0.4, 1, 2, 3, 5, 10 mM
Colchicine	COL	64–86-8	DMSO	0.0125, 0.025, 0.05, 0.1, 0.2, 0.3 μM
2-Deoxy-D-Glucose	2DG	154–17-6	Media	0.3125, 0.625, 1.25, 2.5, 5, 10 mM
Sodium Ascorbate	ASC	134–03-2	Media	0.1, 0.4, 1, 2, 4, 10 mM
Sodium Chloride	NaCl	7647-14-5	Media	0.5, 1, 2.5, 5, 7.5, 10 mM

*indicates a cytotoxic concentration (< 40% RS) that was subsequently eliminated from the analysis; concentrations that are underlined were used for gene expression analysis (a low, mid and high concentration were selected based on criteria in Buick et al. (2015) and Buick et al. (2017) using the % RS and % MN data).

## Materials and methods

### Chemicals

Test chemicals were purchased from Sigma-Aldrich (St. Louis, Missouri, USA) for exposures in human cryopreserved No-Spin HepaRG™ cells (Triangle Research Labs (TRL), Durham, North Carolina, USA; acquired by Lonza Bioscience). The test chemical information, the corresponding vehicle control, and the concentrations tested are presented in Table 1. The chemical exposures in HepaRG™ cultures and the paired high-content flow cytometry data were conducted at Integrated Laboratory Systems, Inc. (ILS; Research Triangle Park, Durham, North Carolina, USA).

### HepaRG™ cell culture and chemical exposures

Human HepaRG™ cells were cultured according to a method adapted from Jossé et al. for use in a slide-based in vitro MN assay [41]. Differentiated HepaRG™ cells were seeded into collagen-coated wells at approximately 1.0–1.75 × 10^5^ viable cells per well in 12-well plates in TRL’s Thawing and Plating Medium for 24 h, then switched to TRL’s Pre-Induction/Tox medium for cell maintenance and treatment. The 12-well plate format was chosen in order to provide sufficient numbers of cells per replicate for RNA extraction and the MN assay without the need to pool wells. Cells were incubated for 7 days following seeding to allow the cells to regain peak metabolic function [34], then they were treated with six concentrations of each test chemical and refreshed with media and test article daily for 3 days (i.e. 3-day repeat exposures; 0 h, 24 h, and 48 h). A multiple exposure procedure was selected to allow for a more gradual induction of metabolic activity that led to more effective bioactivation of certain chemicals, specifically cyclophosphamide [56, 57]. Seven hours following the third treatment (55 h total exposure time), a subset of cells were detached using TrypLE (Waltham, MA), washed with 1X phosphate buffered saline, pelleted, flash-frozen and stored at − 80 °C for RNA extraction and subsequent whole transcriptome profiling. This sampling time was selected as it most aligned with the optimized protocol for use of the TK6 cell line in the presence of rat liver S9, in which a 4 h chemical exposure was followed by a 3 to 4 h recovery time for optimal TGx-DDI performance. Chemical treatments continued for MN frequency testing for a full 24 h following the last treatment (e.g. 3-day repeat exposures for 24 h each; 72 h total treatment time). Test articles were then removed, media was refreshed and the cells were stimulated with human Epidermal Growth Factor-1 (hEGF) for a further 72 h to induce cell division (i.e., 144 h total time following the last chemical exposure). hEGF-1 (Cell Signaling Technology, Danvers, MA) was applied to the cultures at 200 ng/mL immediately following chemical removal and media refreshment, and again 48 h later. The 3-day mitogen stimulation was found to increase the cell population by approximately 2.3-fold. All experiments were run in duplicate for the MN assay and in triplicate for RNA extraction (RNA-Seq was run as a pooled sample for each condition), with concurrent media and vehicle controls. Chemical concentrations were based on previous work with these chemicals in HepaRG™ and other cells at ILS (data not shown) and on literature searches.

### In vitro MicroFlow® MN assay

The flow cytometry-based cytotoxicity and MN assay was performed using the In Vitro MicroFlow® kit (Litron Laboratories, Rochester, New York, USA). Sample preparation, staining and other methods were performed according to the Instructional Manual provided with the kit. Data were collected using a Becton-Dickinson FACSCalibur 2 laser 4-color instrument. Unless precluded by cytotoxicity, 20,000 (± 2000) cells were analyzed to determine relative survival (% RS) and the MN frequency (% MN). A detailed description of the methods is outlined in Buick et al. [53]. In brief, % RS was determined using intact viable nuclei-to-bead ratios in exposed versus control cells by spiking in counting beads to the cell suspensions to function as the internal standards. MN induction was measured simultaneously using the double staining procedure. The RS and MN data were analyzed using generalized estimating equations (GEEs) as outlined in Yauk et al. [54]. Briefly, a normal distribution for the RS data and a binomial distribution for MN data were assumed for the error terms. The geepack library in R was used for this analysis. GEEs only require specification of the first two moments, the mean and the variance. In the MN analysis, a log link function was used. The results were then back transformed to the original scale using the delta method. MN induction was considered to be positive if the MN frequency was statistically significant and at least twofold above matched vehicle controls.

### Total RNA extraction

Total RNA was extracted from exposed and control HepaRG™ cell pellets (*n* = 3) using the Qiagen RNeasy Mini kit (Qiagen, Toronto, Ontario, Canada) with an on-column DNase I digestion, according to the supplier’s protocol. Purified RNA was quantified and assessed for quality with a NanoDrop® ND-1000 spectrophotometer and an Agilent 2200 TapeStation. High quality RNA was used for gene expression analysis (A260/280 ≥ 2.0 and RIN^e^ ranging from 8.3 to 10).

### Library preparation and AmpliSeq whole transcriptome sequencing

Three concentrations (low, mid, high) were selected for gene expression analysis (Table 1) based on the % RS flow cytometry analysis [53, 58]. The top concentration selected ensured that RS was greater than 40% and then concentrations were scaled down from there. In the absence of cytotoxicity, a top concentration of 10 mM was selected.

The Ion AmpliSeq Transcriptome Human Gene Expression Kit (ThermoFisher Scientific, USA) was used to generate libraries from exposed and control HepaRG™ cells according to the manufacturer’s instructions. Briefly, RNA was pooled in equal amounts from all three samples in each treatment group, then 50 ng of the pooled total RNA samples were reverse transcribed to cDNA using a random priming approach with the SuperScript® VILO™ cDNA synthesis kit. Following 11 cycles of amplification of more than 20,000 human RefSeq transcripts (18,574 mRNAs and 2228 non-coding RNAs) using AmpliSeq primers, the resulting amplicons were treated with FuPa reagent to partially digest the primers and to phosphorylate the amplicons. The amplicons generated for each sample pool were then ligated to unique barcode adapters, which were purified using SPRIselect reagent (Beckman Coulter, Brea, California, USA) to perform a magnetic bead-based DNA clean up method. Libraries were then quantified by TaqMan® qPCR using the Ion Library Quantitation kit, normalized to 200 pM, and pooled in equal amounts for multiplex sequencing. The quantified barcoded libraries were diluted to 50 pM for template preparation and chip loading using the Ion Chef™ Instrument for sequencing using the Ion Proton™ sequencer with Ion PI™ Hi-Q™ Sequencing 200 kits and Ion PI™ Chips (V3).

### Read alignment analysis

Raw sequencing data were analyzed and aligned to the reference genome (Human genome Hg 19) using the Ion Torrent Suite software (v5.04) for the Ion Proton. AmpliSeq sequencing data were analyzed using the ampliSeqRNA plugin available through the Ion Torrent server. This plugin uses the Torrent Mapping Alignment Program (TMAP) that has been optimized for Ion Torrent sequencing data and is capable of aligning the raw sequencing reads to a custom reference sequence set that contains all of the human transcripts represented by the AmpliSeq kit.

### Statistical and bioinformatic analyses

Sequencing data are accessible in the National Centre for Biotechnology Information (NCBI) Gene Expression Omnibus (GEO) database under accession number GSE136009. The pooled library was sequenced on a total of five Ion PI™ Chips (V3). All chips were monitored for Ion Sphere Particle (ISP) loading, enrichment and polyclonality, as well as read length and alignment (coverage and quality). Reads from the five chips were pooled and the raw sequencing data were analyzed using the Ion Torrent wizard for pooling samples with identical barcodes from multiple runs. Pooled samples had an average of 11 M valid reads according to the AmpliSeq plugin. Quality assurance and quality control parameters generated by the plugin, included the percentage of reads on target (average: 94.16%; standard deviation 0.7%), the percentage of detected transcripts (average: 61%; standard deviation 1%), and the log2 reads-per-million (RPM) correlation plots (a measure of each gene’s RPM correlation between sample pairs), which revealed no correlation below 0.97. There was no additional normalization applied and no differential gene expression analysis was conducted.

In depth information regarding statistical and bioinformatic analyses for the TGx-DDI biomarker have been previously published [54, 58]. To summarize, the error-weighted average for each biomarker gene was produced by merging AmpliSeq probe ID read counts for the same gene symbol. Hierarchical cluster analysis was done using the hclust function in R (www.r-project.org). In the pamr function of R (www.bioconductor.org), class predictions (DDI vs. non-DDI) were achieved using the Nearest Shrunken Centroids (NSC) method [59], as has been described previously [51, 53, 54, 58]. Briefly, the standardized centroid (SC) was calculated by applying the NSC method for DDI and non-DDI chemicals in the training set and is the mean expression level for each gene in a class divided by its within-class standard deviation. For each DDI and non-DDI chemical, the SC is shrunken in the direction of the overall centroid to create the NSC. Samples were then classified by comparing their gene expression profile to the class of NSCs and then assigned to a class closest to it in squared distance so that the likelihood of class membership was greater than 0.90 [51].

Three different analyses were completed to classify the test chemicals using the TGx-DDI biomarker, including NSC probability analysis (PA; visualized by heatmaps), principal component analysis (PCA), and 2-dimensional hierarchical clustering (2 DC), as previously described [52]. PCA was completed using the prcomp function in R [60], where the training set data [51] was used to approximate the principal components. The PCA loadings obtained from this analysis were applied to the data generated with the ten test chemicals. A scatterplot with data from the training set and the ten test chemicals was generated to visualize the results. Hierarchical cluster analysis was conducted using Euclidean distances with average linkage using the hclust function [61] in the R software. The classification strategy was as follows: if a chemical results in a positive call in any one of three classification analyses (NSC heatmap, PCA, or 2 DC), it was classified as DDI; while a chemical was classified as non-DDI if it did not lead to a positive call in any of the three analyses [54].

## Results

Human HepaRG™ cells were exposed to increasing concentrations of 10 chemicals, five of which are well-characterized for their ability to cause DNA damage. These samples were analyzed by flow cytometry to assess relative survival and MN frequency, and by RNA-Seq to detect the DNA damage response using the TGx-DDI biomarker.

### Relative survival and micronucleus frequency

**Fig. 1:**
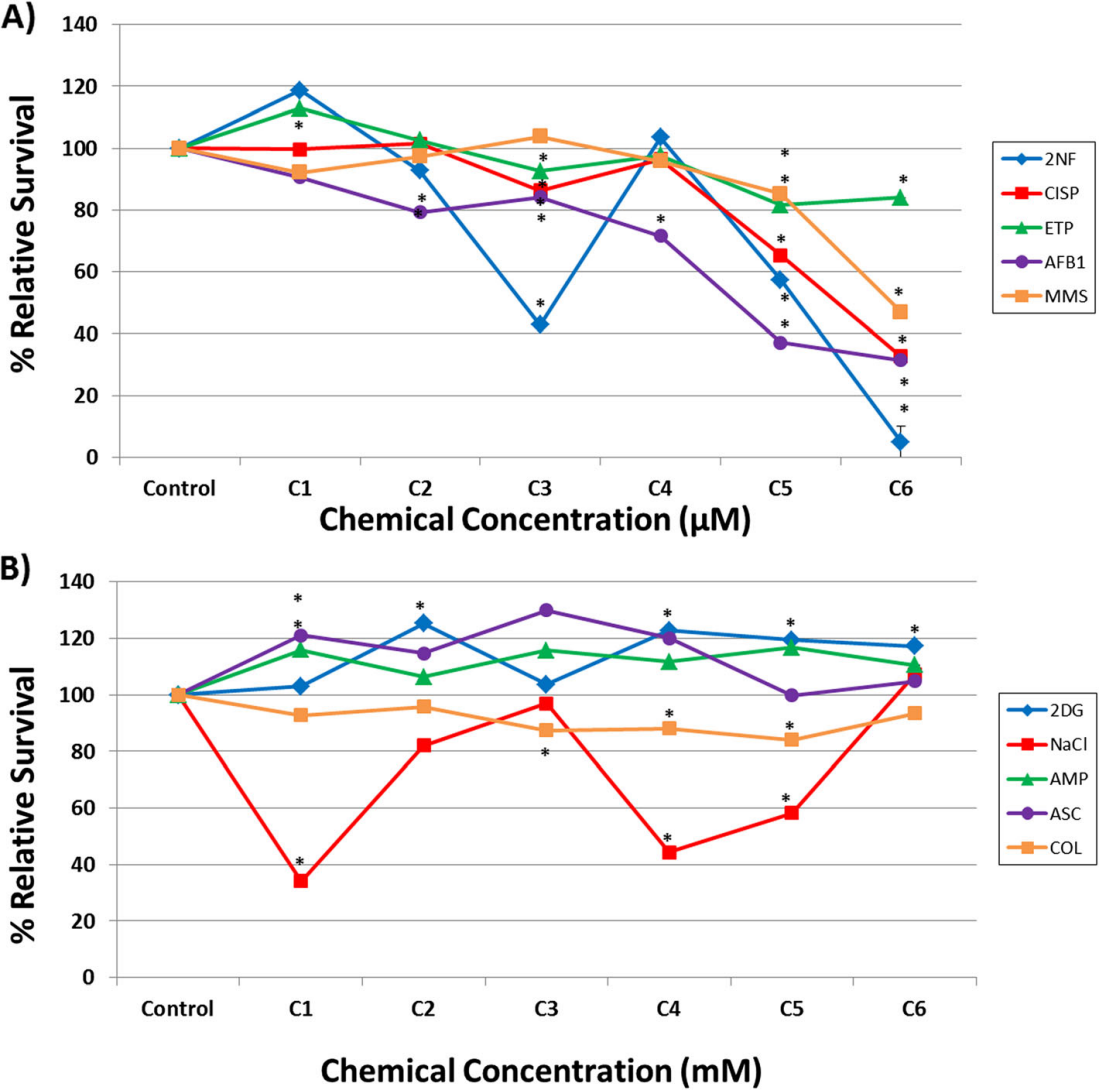
Cytotoxicity assessment in human HepaRG™ cells following exposure to: (**a**) DDI chemicals in μM concentrations; and (**b**) non-DDI chemicals in mM concentrations (except COL, which was in μM) using the In Vitro MicroFlow® assay (Litron Laboratories). See Table 1 for specific concentrations (C1 = lowest concentration and C6 = highest concentration). Percent relative survival is depicted 96 h following the last exposure (*n* = 2). DDI chemical abbreviations: 2-nitrofluorene (2NF), cisplatin (CISP), etoposide (ETP), aflatoxin B1 (AFB1), and methyl methanesulfonate (MMS). Non-DDI chemical abbreviations: 2-deoxy-D-glucose (2DG), sodium chloride (NaCl), ampicillin trihydrate (AMP), sodium ascorbate (ASC), and colchicine (COL). Control represents the vehicle control (DMSO for 2NF, CISP, ETP, AFB1, and COL; water for MMS; media for 2DG, NaCl, AMP, and ASC). Error bars depict standard error, but are too small to see in all but one data point. * *P* < 0.05 compared to the vehicle control

**Fig. 2:**
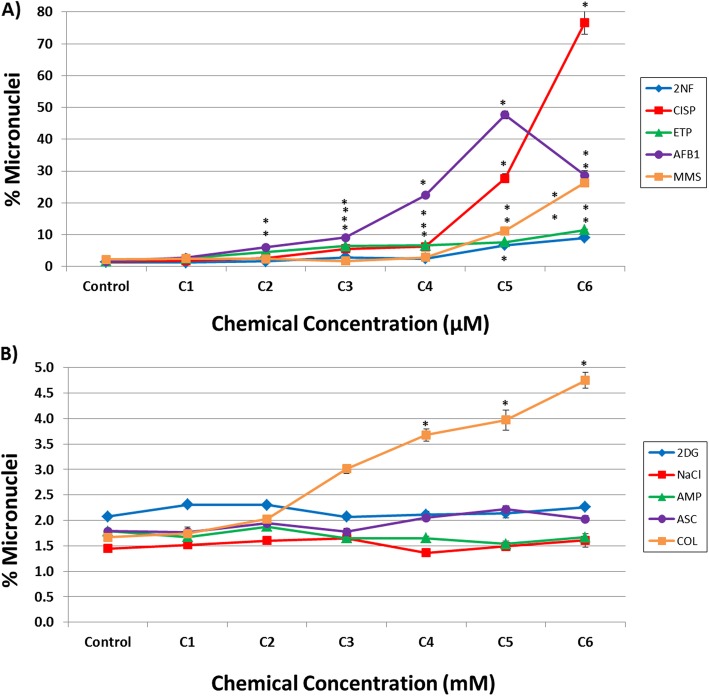
Measurement of MN frequency in human HepaRG™ cells following exposure to: (**a**) DDI chemicals in μM concentrations; and (**b**) non-DDI chemicals in mM concentrations (except COL, which was in μM) using the In Vitro MicroFlow® assay (Litron Laboratories). Percentage of MN induction is depicted 96 h following the last exposure (*n* = 2). See Table 1 for specific concentrations (C1 = lowest concentration and C6 = highest concentration). DDI chemical abbreviations: 2-nitrofluorene (2NF), cisplatin (CISP), etoposide (ETP), aflatoxin B1 (AFB1), and methyl methanesulfonate (MMS). Non-DDI chemical abbreviations: 2-deoxy-D-glucose (2DG), sodium chloride (NaCl), ampicillin trihydrate (AMP), sodium ascorbate (ASC), and colchicine (COL). Control represents the vehicle control (DMSO for 2NF, CISP, ETP, AFB1, and COL; water for MMS; media for 2DG, NaCl, AMP, and ASC). Error bars depict standard error, but are too small to see for many data points. * P < 0.01 compared to the vehicle control

The In Vitro MicroFlow® data, collected following repeated chemical exposures in human HepaRG™ cells, are presented in Figs. 1 and 2. Note that these figures display the full concentration-response of In Vitro MicroFlow® data for all ten test chemicals. Additional file 1 and Additional file 2 only contain the % RS and % MN data for the concentrations selected for RNA-Seq analysis, respectively (described as low, mid, and high concentrations for simplicity; also shown in Table 1), rather than all concentrations tested. Overall, the DDI chemicals (AFB1, CISP, ETP, MMS, and 2NF) caused a concentration-related decline in cell survival (Fig. 1a). In contrast, three of the non-DDI chemicals did not cause any notable cytotoxicity (% RS > 80%) up to 10 mM. NaCl showed some decline in % RS at several concentrations when tested up to 10 mM. Note that colchicine was tested in the μM concentration range (Fig. 1b), as these concentrations were effective in inducing aneugenicity.

A concentration-related increase in % MN was also observed for all DDI compounds (Fig. 2a). ETP and AFB1 induced statistically significant increases in % MN at the top five concentrations tested (C2-C6). MN induction was observed for the top four concentrations of 2NF and CISP (C3-C6), and MMS induced MN at the top two concentrations tested (C5 and C6). The fold changes in % MN over vehicle control observed for the highest, non-cytotoxic concentrations of each DDI chemical were as follows: 4.9-fold for 2NF, 7.1-fold for ETP, 11.8-fold for MMS, 17.8-fold for CISP, and 28.6-fold for AFB1, respectively. No MN induction was observed for the non-DDI chemicals tested, except for COL, which induced MN at the top three concentrations tested (C4-C6), and resulted in a 2.9-fold increase over vehicle control at the highest concentration (Fig. 2b). This response was expected as colchicine is an aneugen that affects microtubule assembly and inhibits tubulin polymerization [62, 63]. It is well established that the MN assay detects both structural and numerical chromosomal alterations in a cell [64–66].

### TGx-DDI biomarker analysis

**Fig. 3:**
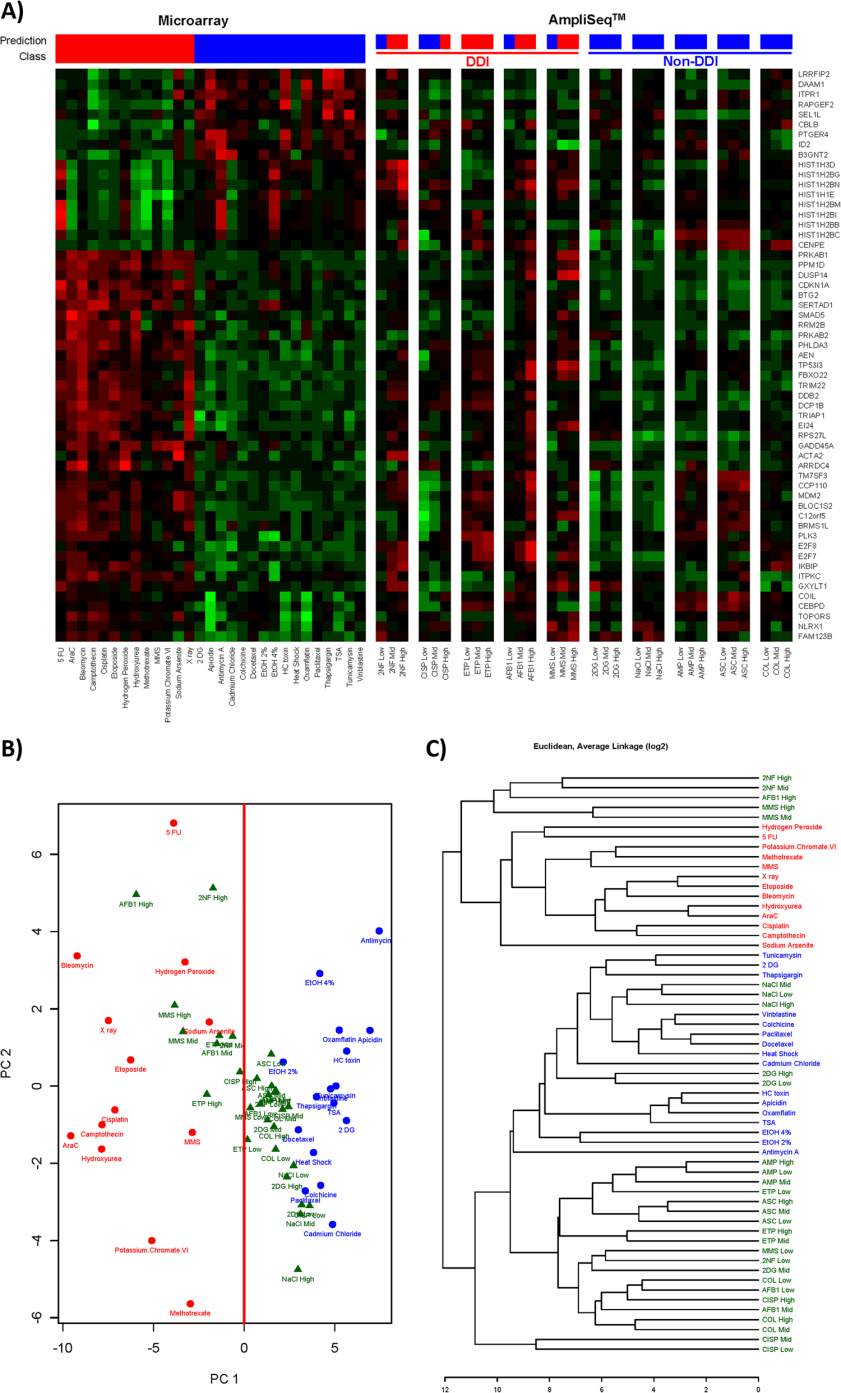
(**a**) The heatmap on the left depicts the responses of the TGx-DDI biomarker genes in the 28 reference chemicals used to generate it by DNA microarray analysis in TK6 cells, and the test chemicals assessed with AmpliSeq in HepaRG™ cells are shown in the subsequent columns. The labels on the far right hand side are Gene Symbols corresponding to the GenBank accession numbers for the biomarker genes. The color scale indicates fold changes relative to control: up-regulated genes are in red, down-regulated genes in green, and genes exhibiting no changes relative to controls are in black. Predictions of DDI/non-DDI and NSC classification probabilities for all treatment conditions are shown using red (DDI) and blue (non-DDI) bars above each heatmap. (**b**) Principal component analysis using the TGx-DDI biomarker for TK6 cells exposed to the training set of chemicals (red text = DDI training set; blue text = non-DDI training set) and for HepaRG™ cells exposed to 10 test chemicals at low, mid, and high concentrations 7 h following the last exposure (green text = replicates of test agent). The line drawn at 0 on the PCA plot divides the DDI and non-DDI agents and was used for classification. (**c**) Hierarchical clustering of the chemicals with TGx-DDI, with color coding as in panel B. The main branch on the dendrogram separates the DDI and non-DDI agents and was used for classification of the test agent

The TGx-DDI genomic biomarker was used to classify the 10 test chemicals as DDI or non-DDI using AmpliSeq whole transcriptome sequencing. Figure 3 depicts the TGx-DDI classification results for all chemicals. Three separate analyses, including NSC Probability Analysis (PA; Fig. 3a), PCA (Fig. 3b), and 2 DC (Fig. 3c) were used to classify the chemicals. A chemical that rendered a positive call in one or more analyses was considered to be DDI; whereas, a chemical that rendered a negative call in all three analyses was considered to be non-DDI. The TGx-DDI genomic biomarker accurately classified all five DDI compounds as DNA damage-inducing. All three concentrations of ETP, the mid and the high concentration of 2NF, AFB1, and MMS, and the high concentration of CISP all classified as DDI based on the combined PA, PCA, and 2 DC analysis (Table 2). The TGx-DDI biomarker accurately classified all five non-DDI test chemicals as non-DDI at the low, mid, and high concentrations in all three analyses (Fig. 3 and Table 2).

**Table 2:** MN frequency and TGx-DDI classification for test chemicals using NSC probability analysis, principal component analysis, and 2-dimensional clustering

Test Chemical	MN Induction			Overall TGx-DDI Classification			PA Classification			PCA Classification			2 DC Classification		
	Low	Mid	High	Low	Mid	High	Low	Mid	High	Low	Mid	High	Low	Mid	High
DDI Chemicals															
Aflatoxin B1	+	+	+	Non-DDI	DDI	DDI	Non-DDI	DDI	DDI	Non-DDI	DDI	DDI	Non-DDI	Non-DDI	DDI
Cisplatin	–	+	+	Non-DDI	Non-DDI	DDI	Non-DDI	Non-DDI	DDI	Non-DDI	Non-DDI	DDI	Non-DDI	Non-DDI	Non-DDI
Etoposide	+	+	+	DDI	DDI	DDI	DDI	DDI	DDI	Non-DDI	DDI	DDI	Non-DDI	Non-DDI	Non-DDI
Methyl methanesulfonate	–	+	+	Non-DDI	DDI	DDI	Non-DDI	DDI	DDI	Non-DDI	DDI	DDI	Non-DDI	DDI	DDI
2-Nitrofluorene	–	+	+	Non-DDI	DDI	DDI	Non-DDI	DDI	DDI	Non-DDI	DDI	DDI	Non-DDI	DDI	DDI
Non-DDI Chemicals															
Ampicillin Trihydrate	–	–	–	Non-DDI	Non-DDI	Non-DDI	Non-DDI	Non-DDI	Non-DDI	Non-DDI	Non-DDI	Non-DDI	Non-DDI	Non-DDI	Non-DDI
Colchicine	–	+	+	Non-DDI	Non-DDI	Non-DDI	Non-DDI	Non-DDI	Non-DDI	Non-DDI	Non-DDI	Non-DDI	Non-DDI	Non-DDI	Non-DDI
2-Deoxy-D-Glucose	–	–	–	Non-DDI	Non-DDI	Non-DDI	Non-DDI	Non-DDI	Non-DDI	Non-DDI	Non-DDI	Non-DDI	Non-DDI	Non-DDI	Non-DDI
Sodium Ascorbate	–	–	–	Non-DDI	Non-DDI	Non-DDI	Non-DDI	Non-DDI	Non-DDI	Non-DDI	Non-DDI	Non-DDI	Non-DDI	Non-DDI	Non-DDI
Sodium Chloride	–	–	–	Non-DDI	Non-DDI	Non-DDI	Non-DDI	Non-DDI	Non-DDI	Non-DDI	Non-DDI	Non-DDI	Non-DDI	Non-DDI	Non-DDI

For MN Induction, a ‘+’ sign indicates a statistically significant induction of MN and at least a two-fold change over vehicle controls and a ‘-‘sign indicates that there was no statistically significant induction of MN. For the TGx-DDI classification results, DDI represents a DNA damage-inducing classification; whereas, non-DDI represents a non-DNA damage-inducing classification. The ‘overall’ DDI call requires a DDI call in at least one of the three analyses (PA, 2 DC, PCA), whereas a non-DDI is non-DDI in all of these analyses

A full summary of the MN and TGx-DDI results is shown in Table 2. MN induction was considered to be positive if the MN frequency was statistically significant (*p* < 0.01 compared to vehicle controls) and at least two-fold higher than the matched controls. Overall, classification as DDI or non-DDI by TGx-DDI was concordant with MN calls and expectations for each chemical, though there were slight discrepancies in the concentrations at which these calls were made for these two assays.

## Discussion

Although genetic toxicity testing is not routinely conducted in HepaRG™ cells, this progenitor cell line is gaining more traction for this purpose [47, 67–69] as these cells can differentiate into hepatocyte- and cholangiocyte-like cells that express human-relevant levels of Phase I and Phase II metabolic enzymes, are easily accessible, and are stable in culture [33–35, 39, 49]. We measured MN frequency by flow cytometry in combination with the TGx-DDI biomarker response by RNA-sequencing to evaluate the application of these tests run in parallel in HepaRG™ cells using 10 genotoxic and non-genotoxic chemicals. A concentration-related decrease in % RS and a concomitant increase in MN frequency was observed for DDI chemicals using our experimental design. In addition, the non-DDI agent colchicine induced a significant increase in % MN as expected, as it is an aneugen. The remaining non-DDI chemicals did not induce MN and generally did not impact % RS up to 10 mM (note: colchicine was tested in the μM range). The TGx-DDI biomarker also correctly classified all test compounds; ETP was classified as DDI at all three concentrations tested, 2NF, AFB1, and MMS at the mid and high concentrations, and CISP at the high concentration only; all non-DDI chemicals were correctly classified at all concentrations. We achieved the expected MN and DDI classifications for the pro-genotoxicants (2NF and AFB1), indicating acceptable biotransformation of these compounds to reactive genotoxic metabolites. In addition, agents not requiring metabolic activation (CIS, ETP, MMS, COL) correctly classified using the TGx-DDI method with this experimental design. Thus, these results demonstrate that these assays worked effectively in 3-day repeat exposure experimental designs in HepaRG™ cells.

Previous studies have demonstrated the utility of HepaRG™ cells for genetic toxicity testing. For example, Jossé et al. [70] assessed the cytotoxicity and genotoxicity of malathion (an insecticide) and isomalathion (an impurity of malathion) either individually or in combination using a 24 h exposure with a hEGF stimulation in HepaRG™ cells. Their results showed that isomalathion is cytotoxic and genotoxic in human liver cells, and that the compounds can display antagonistic and additive effects in combination, where the deleterious effects were dependent on the endpoint and concentration of the test compounds. Recently, Souton et al. [71] studied the genotoxic effects of food contact recycled paperboard extracts in two human hepatic cell lines. They exposed HepaRG™ cells to the paperboard extracts for 24 h without hEGF stimulation and then incubated the cells with cytoB for 28 h. The paperboard extracts from the beginning of the chain did not induce MN, but MN induction was observed following exposure to the end paperboard extracts, indicating that recycled food contact papers can induce genotoxic effects in vitro under these experimental conditions [71]. In another recent study, Allemang et al. [68] demonstrated the utility of the high-throughput MN assay for evaluating the genotoxic potential of 15 pyrrolizidine alkaloids (PAs) in HepaRG™ cells. In this study, a 24 h treatment period with six concentrations of each PA was followed by a 72 h hEGF stimulation. They found marked differences between the most and least potent PA, covering a range of 500x. Overall, despite the fact that the differentiation status of the HepaRG™ cultures differed at the beginning of these experiments (i.e., some studies used cryopreserved terminally differentiated cells and some used fresh cells that were differentiated in the lab over a four week period), the concentrations of DMSO varied, and the chemical exposure and MN protocols in HepaRG™ cells were not conducted in a standardized manner, these studies and our work support that HepaRG™ cells provide a robust model for assessment of the genetic effects using the more traditional MN assay, in addition to the higher-throughput flow cytometry-based version of the assay.

Our study included both pro-genotoxicants (i.e., those requiring metabolic activation to exhibit genotoxic effects), as well as direct-acting substances (no metabolism required), to evaluate the suitability of our experimental design in HepaRG™ cells in genetic toxicology assessment. The suitability of HepaRG™ cells specifically for use in the MN test has previously been confirmed with some adaptations for this cell line [41, 42, 45]. Previous work by Jossé et al. [41] showed that HepaRG™ cells could be adapted to the in vitro MN assay after a single 24 h exposure and a repeat exposure scenario including three chemical treatments with media renewal over 7 days . For our study, we adapted a 3-day repeat exposure. Further adaptations included allowing for hepatocyte enrichment to 80% of the cell population, omitting the cell detachment step following chemical exposure to lower the possibility of cell loss, stimulating cell proliferation with hEGF, and removing the cytochalasin B (cytoB) blocking step of the protocol [41]. Preliminary work with this progenitor cell line at ILS indicated that a repeated exposure design in HepaRG™ cultures enhanced their modeling of metabolic-associated responses more effectively than a single 24 h exposure. This is likely the result of the notable, but diminished metabolic competence of 2D cell cultures unless using very high concentrations of DMSO (MHMET supplement after ~ 10 days of exposure) compared to in vivo levels [39]. By using a 3-day repeated exposure format allowing each test article to induce specific CYP450s, in addition to the adaptations outlined by Jossé et al. [41] above, the use of HepaRG™ cells with the flow cytometry-based in vitro MN assay achieved the expected results using these DDI and non-DDI chemicals.

The MN and TGx-DDI classification results in our study were highly concordant (Table 2) and aligned with well-established knowledge of these test agents, all of which frequently serve as prototypes in assessing the performance of genotoxicity tests and novel cell culture models [72, 73]. Colchicine provides an interesting example of the added-value of the mechanistic information provided by the biomarker; a positive call by MN analysis with a negative call by TGx-DDI indicates that the MN observed may not be occurring through DNA damage. If aneugenicity is thus suspected, additional tests should be performed to investigate further (e.g., MN analysis with CREST staining, or In Vitro MultiFlow® analysis). Using our established criteria for MN calls at each concentration, we noted a marginal increase in sensitivity of the MN assay over the TGx-DDI biomarker (i.e., some low concentrations that classified as non-DDI by the TGx-DDI assay were positive for the MN assay) in HepaRG™ cells. Similarly, in our previous work, we combined the flow cytometry MN assay with TGx-DDI analysis in human TK6 cells in the presence of rat liver S9 to analyze 7 genotoxic and 2 non-genotoxic chemicals [54]. Interestingly, in TK6 cells the opposite was observed in terms of test sensitivity, in that the TGx-DDI biomarker was slightly more sensitive than the MN assay (i.e., some low concentrations were negative for the induction of MN, but classified as DDI by the TGx-DDI assay) [54]. Based on these preliminary studies, it appears that the sensitivities of these two toxicological tests may be cell-type specific and thus will be influenced by the in vitro model of choice. Indeed, Corton et al. [74] demonstrated that the balanced accuracies of the TGx-DDI biomarker vary based on cell line and gene expression technology using an alternative computational method, the Running Fisher test. In this study, the predictive accuracies of the TGx-DDI classifications were determined for TK6 cells and three different liver cell lines (HepaRG^TM^ cells, HepG2 cells, and embryonic stem cell (ESC)-derived hepatocytes) [74]. Using the Running Fisher test, the biomarker had a balanced accuracy of 90% in TK6 and HepaRG™ cells, but the balanced accuracies were not as robust in the other two liver-derived cell lines (80% in ESC-derived hepatocytes and 60% in HepG2 cells), which are less metabolically competent than HepaRG™ cells [74]. Thus, based on previous work of collaborators and others, along with the current study, HepaRG™ cells are a suitable choice of cell line for the MN and TGx-DDI assays. Overall, despite some slight variations in test sensitivities, when run in parallel, the flow cytometry MN assay and TGx-DDI classification using RNA-Seq complement each other well and led to the correct classification of all 10 test compounds.

Observation of the expected responses for the DDI agents within this study confirms an intact response of the p53 pathway in HepaRG™ cells. The TGx-DDI biomarker is enriched in p53-responsive genes that are regulated through this nuclear receptor, and therefore the use of p53-competent cells is a mandatory requirement for this assay [52]. When a positive TGx-DDI classification is rendered, this indicates that sufficient DNA damage has been sustained due to the chemical treatment, which directs the cell to initiate a transcriptional DNA damage response driven by p53 [52]. Indeed, Corton et al. not only confirmed that most TGx-DDI biomarker genes are p53-dependent, but also showed that the biomarker is able to identify a multitude of environmental chemicals, chemotherapeutic drugs, and chemicals that activate p53 [75].

The TGx-DDI genomic biomarker was developed and initially validated using Agilent microarray technology [51–53, 76]. To date, the biomarker has been further validated with several other gene expression technologies, including qPCR [77] and NanoString analysis [52], but validation has been focused on its use in TK6 cells. In this study, we demonstrate that the TGx-DDI biomarker correctly predicts DNA damaging potential using Ion AmpliSeq whole transcriptome gene expression profiling in HepaRG™ cells.

The current study builds on our previous work showing accurate TGx-DDI predictions using Affymetrix DNA microarrays from a publicly available data set in HepaRG™ cells [46, 53]. In that study, HepaRG™ cells were exposed to fifteen compounds (5 genotoxic and 5 non-genotoxic hepatocarcinogens, plus 5 non-carcinogens) for 72 h at concentrations that reduced cell viability by 10% [46]. Only two test chemicals overlapped between that study and our current work: AFB1 and 2NF. Both chemicals rendered positive TGx-DDI calls in each of the studies, but at slightly different concentrations. This highlights the critical importance of concentration selection for TGx-DDI analysis and provides support for using more than one concentration for chemical testing. There are also several noteworthy differences in the experimental design used in the aforementioned study compared to our current work, including the use of fresh versus cryopreserved HepaRG™ cells, a single 72 h exposure versus repeat exposures at 0 h, 24 h, and 48 h with cells collected for RNA extraction 7 h after the last exposure (55 h total exposure time), and concentration selection criteria for the test compounds (IC10 versus > 40% RS). However, despite these differences, our experimental design and that used by Doktorova et al. were equally effective in classifying chemicals as DDI or non-DDI, suggesting that HepaRG™ cells exhibit a robust TGx-DDI response under multiple testing conditions [46]. Moreover, this current experiment provides additional validation that supports TGx-DDI biomarker analysis through modern RNA-sequencing technologies to broaden its application for in vitro genotoxicity testing.

## Conclusions

In summary, this work provides support for the use of HepaRG™ cells with the MN assay in combination with TGx-DDI classification analysis to accurately identify chemicals that cause DNA damage. It also demonstrates how these two genetic toxicology assays may be integrated into a single experimental design. The combination of the flow cytometry-based MN assay with this RNA-Seq approach to TGx-DDI biomarker analysis is a step towards accomplishing a higher-throughput, more integrated genotoxicity testing strategy in metabolically competent human hepatocytes to better inform human health risk assessment.

## Supplementary information

**Additional file 1.** Cytotoxicity assessment in human HepaRG™ cells following exposure to: (A) DDI chemicals in μM concentrations; and (B) non-DDI chemicals in mM concentrations (except COL, which was in μM) using the In Vitro MicroFlow® assay (Litron Laboratories). A low, mid and high concentration were used for AmpliSeq analysis (specific concentrations are underlined in Table 1). Percent relative survival is depicted 96 hr following the last exposure (n = 2). DDI chemical abbreviations: 2-nitrofluorene (2NF), cisplatin (CISP), etoposide (ETP), aflatoxin B1 (AFB1), and methyl methanesulfonate (MMS). Non-DDI chemical abbreviations: 2-deoxy-D-glucose (2DG), sodium chloride (NaCl), ampicillin trihydrate (AMP), sodium ascorbate (ASC), and colchicine (COL). Control represents the vehicle control (DMSO for 2NF, CISP, ETP, AFB1, and COL; water for MMS; media for 2DG, NaCl, AMP, and ASC). Error bars depict standard error, but are too small to visualize. * *P* < 0.05 compared to the vehicle control.

**Additional file 2.** Measurement of MN frequency in human HepaRG™ cells following exposure to: (A) DDI chemicals in μM concentrations; and (B) non-DDI chemicals in mM concentrations (except COL, which was in μM) using the In Vitro MicroFlow® assay (Litron Laboratories). Percentage of MN induction is depicted 96 hr following the last exposure (n = 2). A low, mid and high concentration were used for AmpliSeq analysis (specific concentrations are underlined in Table 1). DDI chemical abbreviations: 2-nitrofluorene (2NF), cisplatin (CISP), etoposide (ETP), aflatoxin B1 (AFB1), and methyl methanesulfonate (MMS). Non-DDI chemical abbreviations: 2-deoxy-D-glucose (2DG), sodium chloride (NaCl), ampicillin trihydrate (AMP), sodium ascorbate (ASC), and colchicine (COL). Control represents the vehicle control (DMSO for 2NF, CISP, ETP, AFB1, and COL; water for MMS; media for 2DG, NaCl, AMP, and ASC). Error bars depict standard error, but are too small to see for many data points. * *P* < 0.01 compared to the vehicle control.

## Data Availability

The datasets generated and analysed in this study are available through the NCBI Gene Expression Omnibus under accession number GSE136009. [https://www.ncbi.nlm.nih.gov/geo/query/acc.cgi?acc=GSE136009].

## Abbreviations

2 DC: 2-dimensional hierarchical clustering
2DG: 2-Deoxy-D-Glucose
2NF: 2-Nitrofluorene
AFB1: Aflatoxin B1
AMP: Ampicillin Trihydrate
ASC: Sodium Ascorbate
CISP: Cisplatin
COL: Colchicine
DDI: DNA damage-inducing
ETP: Etoposide
HESI: Health and Environmental Sciences Institute
HHRA: Human Health Risk Assessment
MMS: Methyl methanesulfonate
MN: micronucleus
MOA: Mode of Action
NaCl: Sodium Chloride
NSC: Nearest Shrunken Centroids
PCA: Principal Component Analysis
RS: Relative survival
TGx: Toxicogenomics
