# Supplementary figures and images for: Flow cytometric micronucleus assay and TGx-DDI transcriptomic biomarker analysis of ten genotoxic and non-genotoxic chemicals in human HepaRG™ cells
Source: Genes Environ. 2020 Feb 4;42:5. doi: 10.1186/s41021-019-0139-2 (PMC7001283; doi:10.1186/s41021-019-0139-2)

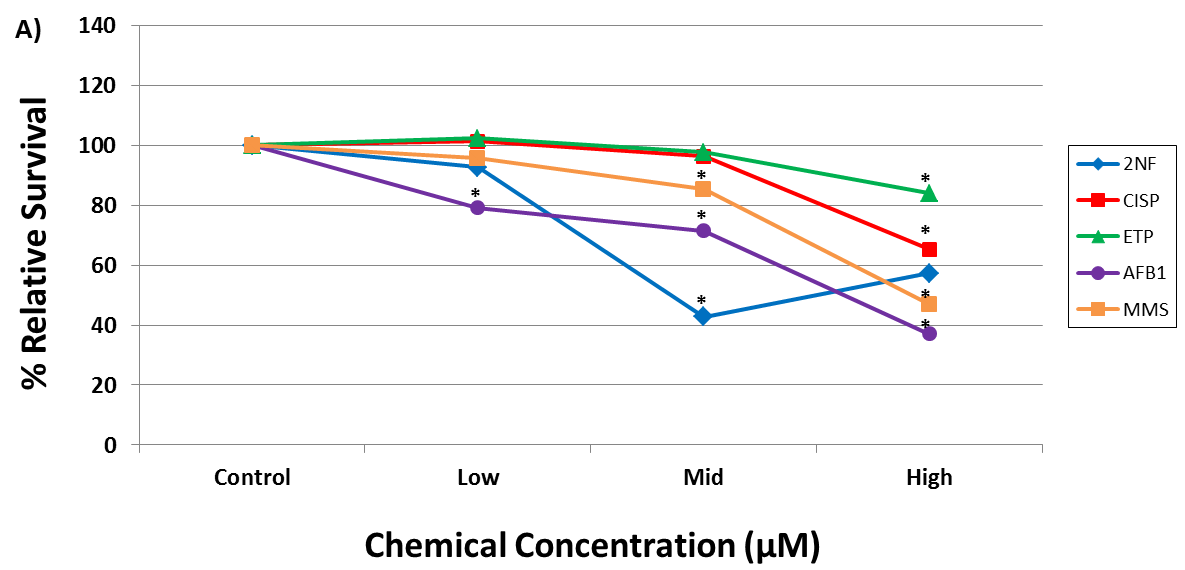


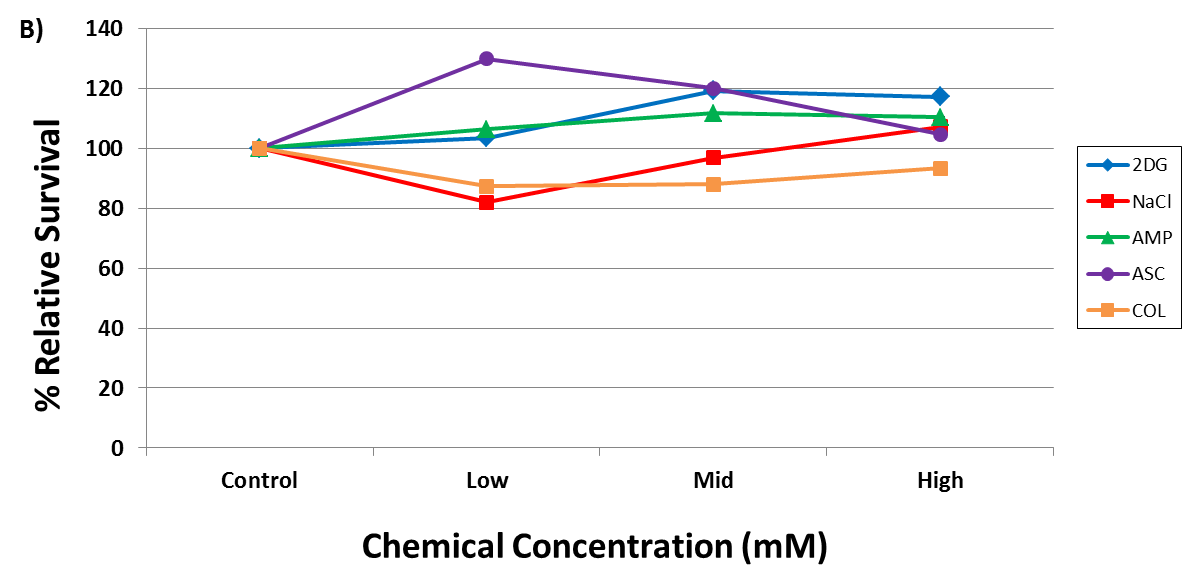

Supplement: Supplementary file 1 — Additional file 1. Cytotoxicity assessment in human HepaRG™ cells following exposure to: (A) DDI chemicals in μM concentrations; and (B) non-DDI chemicals in mM concentrations (except COL, which was in μM) using the In Vitro MicroFlow® assay (Litron Laboratories). A low, mid and high concentration were used for AmpliSeq analysis (specific concentrations are underlined in Table 1). Percent relative survival is depicted 96 hr following the last exposure (n = 2). DDI chemical abbreviations: 2-nitrofluorene (2NF), cisplatin (CISP), etoposide (ETP), aflatoxin B1 (AFB1), and methyl methanesulfonate (MMS). Non-DDI chemical abbreviations: 2-deoxy-D-glucose (2DG), sodium chloride (NaCl), ampicillin trihydrate (AMP), sodium ascorbate (ASC), and colchicine (COL). Control represents the vehicle control (DMSO for 2NF, CISP, ETP, AFB1, and COL; water for MMS; media for 2DG, NaCl, AMP, and ASC). Error bars depict standard error, but are too small to visualize. * P < 0.05 compared to the vehicle control. [file 41021_2019_139_MOESM1_ESM.docx]

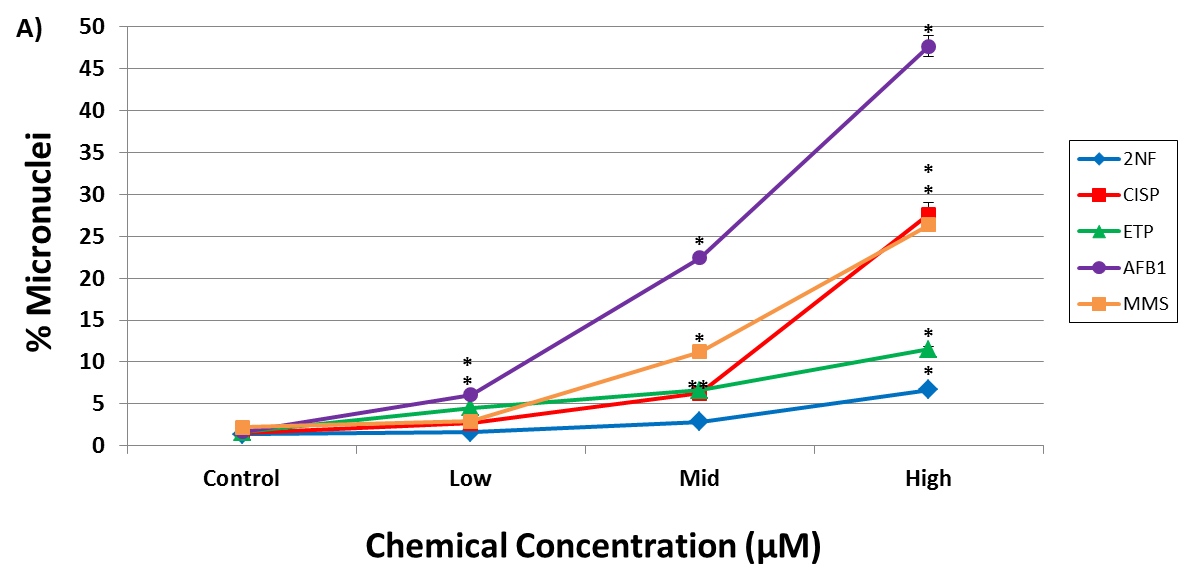


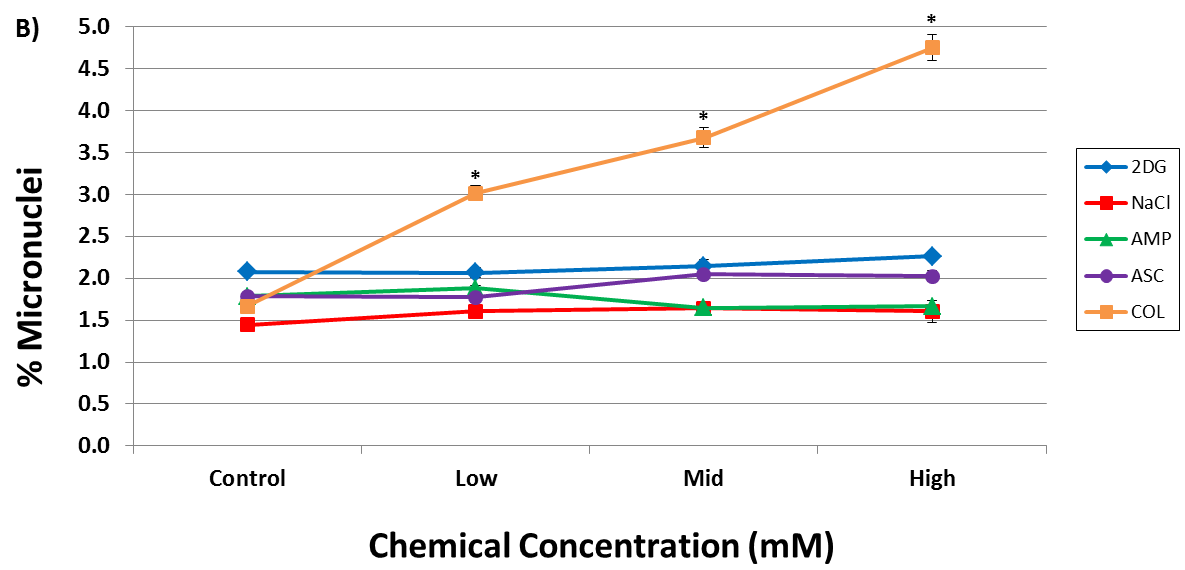

Supplement: Supplementary file 2 — Additional file 2. Measurement of MN frequency in human HepaRG™ cells following exposure to: (A) DDI chemicals in μM concentrations; and (B) non-DDI chemicals in mM concentrations (except COL, which was in μM) using the In Vitro MicroFlow® assay (Litron Laboratories). Percentage of MN induction is depicted 96 hr following the last exposure (n = 2). A low, mid and high concentration were used for AmpliSeq analysis (specific concentrations are underlined in Table 1). DDI chemical abbreviations: 2-nitrofluorene (2NF), cisplatin (CISP), etoposide (ETP), aflatoxin B1 (AFB1), and methyl methanesulfonate (MMS). Non-DDI chemical abbreviations: 2-deoxy-D-glucose (2DG), sodium chloride (NaCl), ampicillin trihydrate (AMP), sodium ascorbate (ASC), and colchicine (COL). Control represents the vehicle control (DMSO for 2NF, CISP, ETP, AFB1, and COL; water for MMS; media for 2DG, NaCl, AMP, and ASC). Error bars depict standard error, but are too small to see for many data points. * P < 0.01 compared to the vehicle control. [file 41021_2019_139_MOESM2_ESM.docx]
